# Sex disparity in adult asthma—A potential immunomodulatory role of let‐7 family microRNAs

**DOI:** 10.1002/clt2.70042

**Published:** 2025-02-28

**Authors:** Carina Malmhäll, Jenny Calvén, Julie Weidner, Kristina Johansson, Patricia Ramos‐Ramírez, Emma Boberg, Linda Ekerljung, Roxana Mincheva, Bright Nwaru, Hannu Kankaanranta, Henric Olsson, Christopher McCrae, Madeleine Rådinger

**Affiliations:** ^1^ Department of Internal Medicine and Clinical Nutrition Krefting Research Centre Institute of Medicine Sahlgrenska Academy University of Gothenburg Gothenburg Sweden; ^2^ Translational Science and Experimental Medicine Research and Early Development Respiratory & Immunology BioPharmaceuticals R&D AstraZeneca Gothenburg Sweden; ^3^ Unit for Lung and Airway Research Institute of Environmental Medicine Karolinska Institutet Stockholm Sweden; ^4^ Bioscience in Vivo Research and Early Development Respiratory and Immunology BioPharmaceuticals R&D AstraZeneca Gothenburg Sweden; ^5^ Faculty of Medicine and Health Technology Tampere University Tampere Finland; ^6^ Department of Respiratory Medicine Seinäjoki Central Hospital Seinäjoki Finland; ^7^ Translational Science and Experimental Medicine Research and Early Development Respiratory & Immunology BioPharmaceuticals R&D AstraZeneca Gaithersburg Maryland USA

**Keywords:** asthma, let‐7, microRNA, sex disparity

## Abstract

**Background:**

Sex differences have been reported in the incidence, prevalence and severity of asthma. Previous findings from animal models have revealed sex‐related differences in inflammatory pathways that may contribute to asthma pathogenesis, but human studies are limited.

**Methods:**

Airway and blood samples (*n* = 55 and *n* = 85 respectively) were collected from adult females and males with asthma and healthy subjects. Type 2 innate lymphoid cells (ILC2s), T helper (Th)2 cells and their expression of IL‐33R/ST2 (ST2L) were evaluated by flow cytometry. IL‐13, thymic stromal lymphopoietin (TSLP), IL‐33 and soluble IL‐33R/ST2 (sST2) were measured by ELISA. Let‐7 miRNA expression in bronchial biopsies was determined by qPCR.

**Results:**

Females with asthma reported more exacerbations and had a higher number of airway eosinophils compared with males with asthma. Bronchial biopsy expression of Let‐7f, Let‐7g and miR‐98 tended to be higher in males with asthma compared with females and inversely correlated with asthma exacerbations. In contrast, increased levels of IL‐13, TSLP and sST2 were found in females with asthma compared with males.

**Conclusion:**

Our study demonstrates different inflammatory signatures between males and females with asthma. Let‐7 miRNAs act as immune modulators by inhibiting the production of IL‐13 and may be an important factor explaining the sex disparity seen in asthma.

## 1 BACKGROUND

Asthma is a heterogeneous disease, typically characterized by chronic airway inflammation and associated with bronchial hyperresponsiveness and airflow obstruction, affecting nearly 300 million people globally.^1^, ^2^ Patients express different phenotypes and display differences in severity, natural history, and response to treatment.^3^ Sex differences have been reported with respect to the incidence, prevalence, and severity of asthma. Asthma is more common in males versus females during childhood, but females have an increased risk of asthma compared to males during and after adolescence.^4^, ^5^ This suggests that changes in sex hormones may play an important role in asthma pathogenesis. Animal models have shown that ovarian hormones increase, whereas testosterone decreases airway inflammation in asthma.^6^ However, the mechanisms for how sex hormones contribute to airway inflammation are not fully understood and studies in humans are lacking.

Though asthma is a heterogeneous disease, a large subset of asthma is characterized by a type 2 immune response. Individuals with type 2 high asthma usually respond well to corticosteroids, but some patients are insensitive to treatment. Allergens, environmental pollutants, viruses, bacteria, and toxins trigger epithelial cells to release alarmin cytokines such as IL‐33 and thymic stromal lymphopoietin (TSLP) which drive the type 2 response. IL‐33 signals via the IL‐33 receptor, serum stimulation‐2 (ST2L), expressed on cells such as group 2 innate lymphoid cells (ILC2s) and type 2 T helper (Th2) cells, both of which produce and release the type 2 cytokines IL‐4, IL‐5, and IL‐13.^7^, ^8^ In addition, IL‐33 functions as a chemoattractant to cells expressing ST2L. Higher levels of ILC2s, both in peripheral blood (PB) and airways, have been reported in asthma patients as compared to controls, but sex differences were not evaluated.^9^, ^10^, ^11^

MicroRNAs (miRNAs) are small non‐coding RNAs that can act as regulators of protein expression by directly binding to their mRNA targets and inhibiting mRNA translation or causing mRNA degradation, thereby modifying cellular behavior. In asthma and chronic obstructive pulmonary disease, miRNAs have been shown to be involved in regulating pathways associated with inflammation, suggesting they are critical regulators of the immune response.^12^ Sex‐related differences in miRNA expression have been attributed to both miRNA enrichment on the X‐chromosome and the effect of sex hormones and may play an important role in mediating sex disparity in disease.^13^, ^14^ However, studies on asthma are currently lacking.

As most sex‐related studies in the asthma field have been performed using animal models, we aimed to uncover factors contributing to sex disparity in adult asthma pathogenesis by analyzing samples from the airways and systemic circulation of well‐characterized individuals with asthma and healthy subjects. In the current study, we found significant differences between males and females with asthma in the number of eosinophils and ILC2s, levels of IL‐13, TSLP, soluble ST2 (sST2) and ST2L expression on ILC2s in the airways. In addition, let‐7f, let‐7g and miR‐98 expression in bronchial tissue tended to be higher in males with asthma compared with females and was associated with asthma control and less frequent exacerbations.

## 2 METHODS

### 2.1 Study participants and sampling

Study participants were mainly invited from the West Sweden Asthma Study, a population‐representative longitudinal study on adult asthma and respiratory health.^15^ All participants volunteered to donate blood samples, and some participants additionally volunteered to undergo bronchoscopy for sampling of bronchial lavage fluid and bronchial biopsies. All participants gave written informed consent to a study protocol approved by the Regional Ethical Committee, Gothenburg, Sweden (no.228‐14). Supporting Information S1: Methods.

### 2.2 Analysis of cells and mediators

**FIGURE 1:**
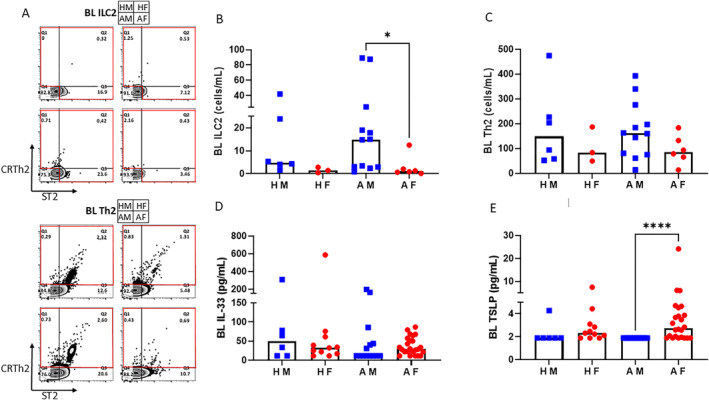
Airway type 2 cells and alarmin levels. (A) Flow cytometry plots show the final gating step for ILC2 (Live, Singlets, CD45+, Lymphocytes, Lin‐, CD127+, ST2+ and/or CRTh2+) and Th2 cells (Live, Singlets, CD45+, Lymphocytes, Lin+, CD4+, ST2+CRTh2+ or CRTh2+) in bronchial lavage samples. For ILC2s and Th2 cells, respectively, HM is shown top left, HF top right, AM bottom left and AF bottom right. Red line indicates the definition of ILC2s and Th2 cells, respectively. (B) Number of ILC2 cells/mL in bronchial lavage fluid. (C) Number of Th2 cells/mL in bronchial lavage fluid (*n* = 3–12/group). (D, E) Levels of IL‐33 and TSLP, respectively, in bronchial lavage fluid (*n* = 6–24/group). Black histogram indicates median value. AF, Asthmatic Females; AM, Asthmatic Males; BL, Bronchial Lavage; HF, Healthy Females; HM, Healthy Males. Kruskal–Wallis test, followed by Dunn's multiple comparison test, was used for comparison between groups. *p < 0.05. ****p < 0.0001.

ILC2s from bronchial lavage cells and peripheral blood mononuclear cells were defined as Lin‐, CD127+, CRTH2+ and/or ST2+ using flow cytometry. Th2 cells were defined as Lin+, CD4+, CRTH2+ST2+ or CRTH2+ST2‐. Representative gating strategy is shown in Figure 1A. Human IL‐33, IL‐13, sST2/IL‐33R and TSLP were analyzed in cell‐free bronchial lavage fluid and serum. Testosterone and estradiol levels were determined in serum samples. Supporting Information S1: Methods and Table S1.

### 2.3 MicroRNA analysis

RNA was isolated from bronchial biopsies. Biopsies from three donors from each group were used in a targeted multiplex miRNA assay for screening. Additional biopsies were used to measure the relative expression of let‐7a‐5p, let‐7f‐5p, let‐7g‐5p and miR‐98‐5p using qPCR. Supporting Information S1: Methods and Table S2.

### 2.4 Statistics

GraphPad Prism version 9.5.0 (GraphPad Software, San Diego, CA, USA) or IBM SPSS statistics version 29 (SPSS Inc., Chicago, IL, USA) were used for statistical analysis. The Mann–Whitney *U* test or Kruskal–Wallis test, followed by Dunn's multiple comparison test, were used for continuous variables. The Chi‐squared test was used for categorical variables. Correlations were performed using Spearman's Rank correlation test. A value of *p* < 0.05 was considered statistically significant.

## 3 RESULTS

### 3.1 Females with asthma report more frequent exacerbations and demonstrate potentiated airway inflammation

In total, 58 individuals with asthma (23 male; 35 female) and 27 healthy controls (13 male; 14 female) participated in this study. The demographic and clinical characteristics of the participants (Table 1) showed that statistically significant differences were found between healthy and asthma subjects. Differences between adult males and females with asthma were found in the number of asthma exacerbations over the last 12 months (*p* = 0.028), asthma medications (*p* = 0.009), Phadiatop sIgE (*p* = 0.037), airway eosinophils (*p* < 0.001) and as expected, the ratio of serum testosterone/estradiol levels (*p* < 0.001). Female participants with asthma had more exacerbations and higher numbers of airway eosinophils compared with male participants with asthma. Males with asthma had higher sIgE values than females. It was more common for females than males to use ICS combined with short‐acting β2‐agonists (SABA), while the use of ICS plus long‐acting β2‐agonists (LABA) with or without other medication, including SABA, was similar between sexes.

**TABLE 1:** Demographic and clinical characteristics of study participants.

	Healthy males	Healthy females	Asthmatic males	Asthmatic females	*p*‐value
No. of participants	13	14	23	35	
Demographic characteristics					
Age (years)	44 (28–63)	46 (29–62)	42 (24–69)	56 (25–73)	0.173
BMI	24 (21–31)	26 (18–33)	27 (21–31)	28 (21–35)	0.070
Current smokers	0	0	0	0	
Asthma phenotype					
Atopy	2 (15.4%)	3 (21.4%)	17 (73.9%)	23 (65.7%)	**0.001**
Age at asthma onset (years)			10 (2–52)	19 (1–58)	0.080
Lung function					
FEV1, predicted (%)	104.3 (78.6–137.2)	101.5 (85.0–121.0)	93.8 (47.1–118.5)^ǂ^	95.0 (73.5–115.0)	**0.025**
FEV1/FVC (%)	84 (72–94)	79 (71–89)	74 (46–90)^ǂǂǂ^	77 (58–88)	**0.001**
FVC (%)	105 (80–121)	112 (96–136)	101 (74–120)	104 (82–143)	0.110
TLCO, predicted (%)	105.3 (73.2–135.0)	103.8 (82.6–132.1)	101.6 (83.1–135.3)	103.6 (83.1–135.4)	0.989
Asthma control and exacerbations					
Controlled			14 (60.8%)	14 (40.0%)	0.232
Partly controlled			7 (30.4%)	13 (37.1%)	
Not controlled			2 (8.7%)	7 (20.0%)	
Asthma exacerbations last 12 months			0 (0–5)	0 (0–7)*	**0.028**
Asthma medication					
ICS+SABA			2 (8.7%)	13 (37.1%)	**0.009**
ICS+LABA			5 (21.7%)	1 (2.8%)	
ICS+LABA+other			16 (69.6%)	21 (60.0%)	
Blood biomarkers					
Phadiatop sIgE (IU/mL)	0.08 (0.03–6.89)	0.06 (0.04–4.53)	7.92 (0.07–101.72)^ǂǂǂ^	0.36 (0.03–70.28)^###,^ *	**0.001**
Blood differential cell count					
Leukocytes (x10^9^/L)	5.3 (3.7–8.1)	5.25 (3.4–7.0)	5.3 (1.4–8.0)	5.3 (3.0–8.2)	0.922
Eosinophils (x10^9^/L)	0.2 (0.07–0.6)	0.15 (0.04–0.5)	0.2 (0.09–0.8)	0.2 (0.04–0.8)	0.977
Neutrophils (x10^9^/L)	2.5 (1.9–4.6)	3.1 (2.0–4.2)	2.9 (1.9–4.4)	2.8 (1.3–5.3)	0.306
Basophils (x10^9^/L)	0 (0–0.1)	0 (0–0.1)	0 (0–0.1)	0 (0–0.1)	0.937
Monocytes (x10^9^/L)	0.4 (0.2–1.4)	0.3 (0.2–0.4)	0.4 (0.2–0.7)	0.4 (0.2–0.8)	0.142
Lymphocytes (x10^9^/L)	1.8 (0.9–3.3)	1.45 (1–3.7)	1.7 (1.4–3.1)	1.9 (1–3.1)	0.300
Bronchial lavage differential cell count	*n* = 6	*n* = 11	*n* = 14	*n* = 22	
Eosinophils (x10^4^/mL)	0.04 (0–0.34)	0.14 (0–0.91)	0 (0–0.5)	0.22 (0–2.77)***	**<0.001**
Neutrophils (x10^4^/mL)	1.23 (0.49–3.03)	0.31 (0.04–3.08)^$^	0.68 (0–2.89)	0.32 (0.02–2.31)	**0.034**
Monocytes (x10^4^/mL)	0.17 (0–1.28)	0.29 (0.15–2.29)	0.19 (0.01–0.43)	0.33 (0.11–3.22)	0.067
Macrophages (x10^4^/mL)	6.93 (4.32–10.74)	7.95 (4.3–11.69)	6.35 (0.4–18.73)	8.32 (2.83–18.65)	0.212
Lymphocytes (x10^4^/mL)	0.76 (0.36–2.00)	0.54 (0.21–2.05)	0.47 (0.02–2.27)	0.59 (0.14–8.3)	0.479
Hormonal factors					
Females on hormonal treatment (*n*)		3 (21.4%)		13 (37.1%)	0.289
Ratio testosterone/Estradiol	84.99 (49.14–99.12); *n* = 6	27.29 (15.53–31.18)^$^; *n* = 3	74.98 (45.64–339.24); *n* = 14	13.78 (6.60–32.94)***; *n* = 13	**<0.001**

*Note*: Data are presented as median value (range), unless otherwise expressed. Kruskal‐Wallis or U‐Mann Whitney test for continuous variables and chi squared test for categoric variables. Significance level is indicated by the following signs. ^$^comparison Healthy Males (HM) versus Healthy Females (HF). ^ǂ, ǂǂǂ^comparison HM versus Asthmatic Males (AM). ^##, ###^comparison HF versus Asthmatic Females (AF). *, ***comparison AM versus AF. A value of *p* < 0.05 was considered statistically significant and is indicated in the table in bold font.

Abbreviations: BMI, Body mass index; FEV1, forced expiratory volume in 1 s; FVC, forced vital capacity; TLCO, transfer factor for carbon monoxide; ICS, Inhaled corticosteroids; SABA, Short‐acting beta‐agonists; LABA, Long‐acting beta‐agonists; Hormonal treatment comprice tablets, patch or contraceptives.

### 3.2 Males and females with asthma display differences in ILC2 numbers and TSLP levels in the airway

Effector cells driving type 2 immunity were evaluated in freshly collected samples. Within the asthma group, higher numbers of airway ILC2s were detected in males than females (*p* = 0.0288, Figure 1B). However, there was no difference in Th2 cell numbers in the airways between males and females (Figure 1C). Furthermore, there was no difference in ILC2s or Th2 cells in blood between males and females (Figure S1A,B). Alarmin cytokines play an important role in asthma as potent regulators of ILC2s and Th2 cells.^7^, ^8^ Thus, we evaluated the levels of IL‐33 and TSLP in cell‐free BL fluid and serum. BL IL‐33 levels were equal in all groups, but within the asthma group, TSLP levels were higher in females compared to males (*p* < 0.0001, Figure 1D–E). No difference was found in serum TSLP between males and females (Figure S1C), and serum IL‐33 levels were below the detection limit.

### 3.3 Altered ST2 levels in females versus males with asthma

**FIGURE 2:**
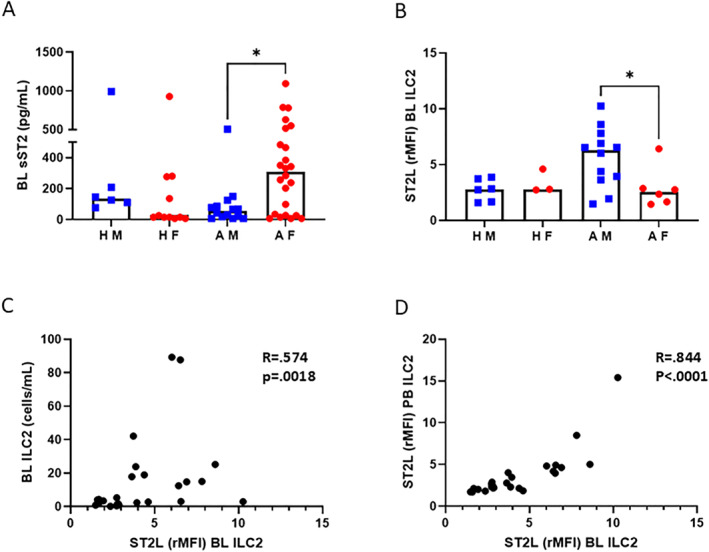
Analysis of soluble ST2 and transmembrane ST2L. (A) sST2 in bronchial lavage fluid (*n* = 6–24/group). (B) Relative median fluorescence intensity (rMFI) of ST2L on ILC2 cells from bronchial lavage (*n* = 3–12/group). (C) Positive correlation between number of ILC2 cells and ST2L expression on ILC2 cells in bronchial lavage. Spearman *R* = 0.574, *p* = 0.002. (D) Positive correlation between ST2L expression on ILC2 cells in blood and airway. Spearman *R* = 0.844, *p* < 0.001. Figures C and D include both males and females. AF, Asthmatic Females; AM, Asthmatic Males; BL, Bronchial Lavage; HF, Healthy Females; HM, Healthy Males; PB, Peripheral Blood. The Kruskal–Wallis test, followed by Dunn's multiple comparison test, was used for comparison between groups. *p < 0.05.

As ST2 is critical for ILC2 regulation, we investigated the relationship between sST2 and transmembrane ST2L. Within the asthma group, females exhibited increased levels of sST2 in bronchial lavage compared with males (*p* = 0.0455, Figure 2A). Conversely, ST2L expression on airway ILC2s was higher in males than in females with asthma (*p* = 0.0320, Figure 2B). On a systemic level, no difference was found in serum sST2 levels or blood ILC2s ST2L expression between the sexes (Figure S1D,E). Interestingly, the number of airway ILC2s correlated positively to ST2L expression on ILC2s in the airways and blood (Spearman *R* = 0.574, *p* = 0.0018 and *R* = 0.844, *p* < 0.0001 respectively, Figure 2C–D). Taken together, our results demonstrate that females with asthma exhibit higher TSLP and sST2 levels in the airways but lower ST2L on ILC2s compared with males with asthma.

### 3.4 Females with asthma have increased levels of IL‐13 in the airway

**FIGURE 3:**
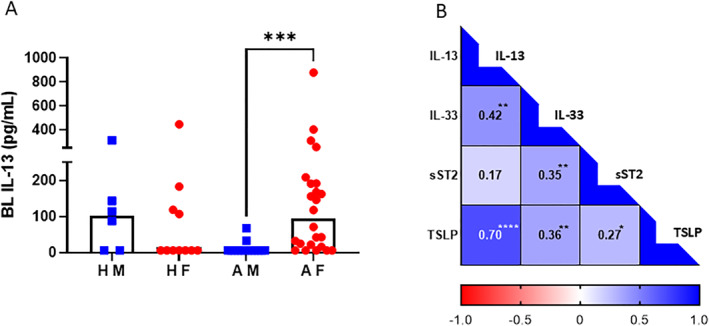
Airway IL‐13 levels in males and females. (A) Levels of IL‐13 in bronchial lavage fluid. Black histogram indicates median value (*n* = 6–24/group). AF, Asthmatic Females; AM, Asthmatic Males; BL, Bronchial Lavage; HF, Healthy Females; HM, Healthy Males. (B) Matrix displaying correlations between BL mediators IL‐13, IL‐33, sST2 and TSLP in all subjects regardless of group (*n* = 55) *p < 0.05, **p < 0.01, ***p < 0.001, ****p < 0.0001. The Kruskal–Wallis test, followed by Dunn's multiple comparison test, was used for comparison between groups. All correlations were performed using Spearman's Rank correlation test.

The downstream effect of alarmins is the induction and release of type 2 cytokines such as IL‐13. The level of IL‐13 in bronchial lavage fluid in asthma was increased in females compared with males (*p* = 0.0007, Figure 3A). To evaluate if there was any association between IL‐13, IL‐33, sST2 and TSLP, we performed a correlation matrix analysis using values for each mediator regardless of group. We found a positive correlation between IL‐13 and both IL‐33 and TSLP but not sST2. However, sST2 levels were positively associated with both IL‐33 and TSLP levels (Figure 3B).

### 3.5 Let‐7 family miRNAs are predicted to target IL‐13

Given that males with asthma were found to have more type 2 cells (ILC2s) but less alarmin cytokines (TSLP) and type 2 cytokines (IL‐13) compared to females, we explored the role of miRNAs as an additional modulator of the immune response. miRNA expression was screened in bronchial biopsies (Figure S2A–I) using NanoString technology. We found that some of the let‐7 family miRNAs tended to be higher in males than females with asthma, which combined with them having a potential inhibitory effect on IL‐13 according to in silico prediction using miRDB, a MicroRNA Target Prediction Database (https://mirdb.org/, September 2020),^16^ implicating them as potential drivers of sex disparity in asthma.

**FIGURE 4:**
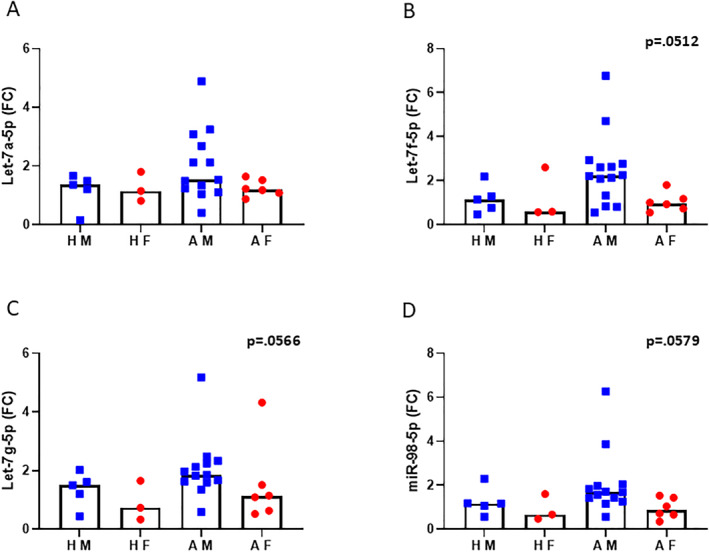
Selected let‐7 family members in bronchial biopsies validated by qPCR. (A‐D) Values of let‐7a‐5p, let‐7f‐5p, let‐7g‐5p, and miR‐98‐5p were normalized to miR‐103‐3p and fold change (FC) was calculated to the mean value of all healthy controls (*n* = 3–12/group). AF, Asthmatic Females; AM, Asthmatic Males; HF, Healthy Females; HM, Healthy Males. The Kruskal–Wallis test was used for comparison between groups.

Eight out of nine let‐7 miRNAs analyzed were similarly rated on IL‐13 binding in the miRDB prediction. Four let‐7 family miRNAs, let‐7a‐5p, let‐7f‐5p, let‐7g‐5p and miR‐98‐5p, were selected for further analysis in additional bronchial biopsy samples using qPCR. Let‐7f, let‐7g and miR‐98 displayed a clear tendency to differ in expression levels (Figure 4B–D) between males and females with asthma.

### 3.6 Asthma control and exacerbations correlate with let‐7 levels in bronchial biopsies

**FIGURE 5:**
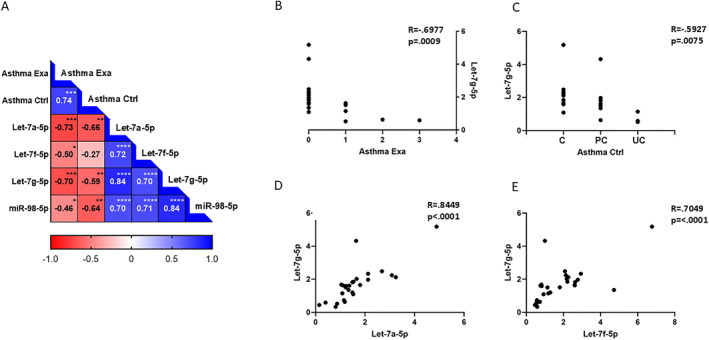
Associations between level of asthma control, number of asthma exacerbations and let‐7 family miRNAs in bronchial biopsies. (A) Matrix displaying negative correlations between expression of let‐7 miRNAs and number of asthma exacerbations over the previous 12 months (Asthma Exa) and level of asthma control (Asthma Ctrl) in subjects with asthma regardless of sex (*n* = 19). (B) Negative correlation between number of asthma exacerbations over the previous 12 months and let‐7g‐5p. Spearman *R* = −0.6977, *p* = 0.0009. (C) Negative correlation between level of asthma control and let‐7g‐5p where C=controlled; PC=partially controlled; and UC=uncontrolled. Spearman *R* = −0.5927, *p* = 0.0075. (D) Positive correlation between let‐7g‐5p and let‐7a‐5p. Spearman *R* = 0.8449, *p* < 0.0001. (E) Positive correlation between let‐7g‐5p and let‐7f‐5p. Spearman *R* = 0.7049, *p* < 0.0001. All correlations included both males and females and were performed using Spearman's Rank correlation test. *p < 0.05, **p < 0.01, ***p < 0.001, ****p < 0.0001.

Intracellular miRNA expression has been shown to be tissue‐ and disease‐specific and therefore has the potential to be used as a prognostic and diagnostic biomarker. GINA 2006 evaluation was used to investigate if the let‐7 family of miRNAs are part of the underlying pathology and have potential impact on asthma control and exacerbations. We performed a correlation matrix analysis using expression levels of the four miRNAs (let‐7a, let‐7f, let‐7g and miR‐98) in bronchial tissue, reported number of exacerbations over the previous 12 months and level of asthma control in subjects with asthma (Figure 5A). We observed that participants reporting less control and more exacerbations showed negative correlations with let‐7 family miRNAs (Figure 5A), indicating a protective role in asthma outlined for let‐7g in Figure 5B–C. Let‐7a, let‐7f, let‐7g and miR‐98 miRNAs correlated positively to each other, suggesting a functional overlap likely linked to their sequence similarity (Figure 5A,D–E).

## 4 DISCUSSION

By analyzing human samples derived from the airways and blood of well‐characterized adult males and females with asthma, this study investigates how cells, mediators and let‐7 family miRNAs may interact to contribute to asthma pathogenesis and the observed sex disparity of the disease. Our samples were obtained from participants with asthma on ICS treatment who reported to not experience symptoms at the time of sampling; we none‐the‐less observed signs of airway inflammation within the asthma group. However, the inflammatory signatures were different between males and females, suggesting a sex disparity in the immune regulation underpinning the pathogenesis of the disease.

Our clinical data revealed that males presented higher Phadiatop sIgE levels, whereas females showed higher numbers of airway eosinophils and more frequent exacerbations within the previous 12 months. However, IgE levels may not only be disease‐related but also sex‐related. Several studies have shown higher IgE levels in males compared to females from childhood through adulthood, even though both sexes generally show decreased levels later in life.^17^ The elevated airway eosinophil counts in females suggest a more inflammatory milieu, which likely contributes to the higher frequency of exacerbations reported among females with asthma.

In our study, males with asthma displayed higher numbers of ILC2s in the airway than females with asthma. This is in contrast with a previous study that reported higher numbers of ILC2s in mild atopic females with asthma at baseline.^18^ However, that study analyzed sputum samples, not bronchial lavage samples, and included only asthma patients with atopy, whereas our study included both atopic and non‐atopic subjects. The same study also investigated a severe asthma group and found no significant differences between the sexes at baseline. Another study reported higher numbers of blood ILC2s in females with asthma compared with males.^19^ Factors such as asthma severity or demographic composition may account for these differences in the number of ILC2s. However, our findings are in line with those of Aw et al., who reported no difference in blood ILC2s between sexes.

Data from animal models demonstrated that ILC2s have higher expression of the androgen receptor compared to other immune cells, suggesting that these cells may be regulated by testosterone.^20^ In contrast, only uterine ILC2s express estrogen receptors,^21^ indicating that estrogen levels may have little impact on ILC2s in other compartments. In experimental animal models, regardless of whether the ILC2s were isolated from inflamed or control tissue, ILC2s from male mice produce less IL‐13 and IL‐5 than ILC2s from female or castrated male mice.^22^, ^23^

The alarmins IL‐33 and TSLP are released from non‐immune cells (such as epithelial and endothelial cells) to signal danger, which activates ILC2s and Th2 cells. Our study showed higher concentrations of TSLP in females with asthma compared to males, whereas no difference was observed for IL‐33. However, IL‐33 exists in reduced and oxidized forms with different bioactivity, and the ELISA method used in the current study is unable to distinguish between these forms of IL‐33.^7^ Our results demonstrated a negative association between sST2 levels and ST2L expression on the surface of ILC2s in the airways. Furthermore, ST2L expression on ILC2s in both the airway and blood was positively associated with ILC2 counts in the airway. Upregulated ST2 (*IL1RL1*) transcripts have been reported in bronchoalveolar lavage ILC2s after segmental allergen challenge, which also resulted in increased numbers of ILC2s in the airway and reduced numbers in the blood.^24^ Moreover, sST2 can function as a decoy receptor for IL‐33 and thereby have an immune regulatory role by sequestering free IL‐33, resulting in less chemoattraction for ST2L bearing cells. Elevated sST2 levels are associated with disease severity in cardiovascular disease. Although elevated serum levels inhibit IL‐33 signaling and are negatively associated with asthma exacerbations, the relationship between sST2 and asthma severity is unclear.^25^

As both elevated TSLP and ILC2 numbers indicate a type 2 response, we measured the type 2 cytokine IL‐13 in bronchial lavage and found that females with asthma exhibited higher levels of IL‐13 than males with asthma. While increased IL‐13 protein expression in females has previously been demonstrated in the lungs of female mice compared to males after allergen challenge,^6^ we are the first to demonstrate this finding in human airway samples. IL‐13 has been shown to promote eosinophil recruitment,^7^, ^8^ which is in line with our observation of higher numbers of airway eosinophils in females with asthma compared to males. We also identified that airway IL‐13 levels were positively associated with IL‐33 and TSLP levels but not significantly with sST2. However, sST2 levels were associated with both IL‐33 and TSLP levels, suggesting a positive relationship between all measured mediators in the airways.

Taken together, our data suggest a stronger type 2 response in females with asthma than in males with asthma. Thus, we hypothesized that additional immune regulatory agents such as miRNAs contribute to this discrepancy. While miRNA profiling in bronchial biopsies revealed a tendency for increased expression of several let‐7 family miRNAs in males with asthma compared with females, this finding was not statistically significant. However, let‐7 miRNAs are predicted to bind to the 3′ UTR region of the *IL1*3 mRNA. The let‐7 family miRNAs have been shown to directly target *IL13* and thus inhibit *IL13* transcription both in animal models and in human cells *in vitro* ^26^, ^27^ Validation of four let‐7 miRNA candidates in additional samples from our cohort confirmed the tendency for increased expression of let‐7f, let‐7g and miR‐98 in males with asthma compared with females with asthma. Using let‐7 mimics, Kumar et al. showed that several members of the let‐7 family can inhibit IL‐13 production in activated primary T cells. They further demonstrated that intranasal delivery of let‐7 mimics reduced the levels of IL‐13 in a murine allergic airway model. Another study demonstrated a negative correlation between miR‐98‐5p and IL‐13 in the serum of patients with asthma and confirmed *IL‐13* to be a target of miR‐98‐5p.^27^ Increased expression of let‐7b in the airways upon house dust mite exposure has been demonstrated in mice.^28^ However, blockade by let‐7b antagomir treatment was ineffective in modulating airway inflammation, potentially due to redundant signaling between multiple members of the let‐7 family.^28^ None of the studies mentioned above reported any data regarding potential sex‐related differences. miRNAs are enriched on the X‐chromosome. Inactivation of one X‐chromosome in females should silence most genes, but it has been shown that genes can escape silencing,^13^ which may cause increased expression of certain miRNAs in some cell types. However, not only X‐chromosome linked miRNAs are differently expressed in males and females. We have previously shown higher expression of miR‐155 in ILCs and Th cells from females compared to males upon various stimuli *ex vivo* ^29^ In this study, two of the let‐7 family members analyzed, let‐7f‐5p and miR‐98‐5p, are located on the X‐chromosome. Thus, it is likely that increased miRNA expression due to chromosomal location is not applicable in this case, as higher levels of these miRNAs would be expected in females.

The observed clinical differences in males and females with asthma prompted us to evaluate whether the levels of let‐7 miRNAs in bronchial biopsies could be associated with the clinical phenotypes of asthma. By analyzing the study participants' levels of asthma control and the number of exacerbations within the previous 12 months, we found a strong negative correlation between let‐7 miRNAs and both asthma control and exacerbations. This suggests that let‐7 miRNAs may play a protective role in asthma, potentially by inhibiting IL‐13.

### 4.1 Strengths and limitations

The strengths of this study are our well‐characterized study participants and the access to paired airway‐ and blood‐derived samples. In contrast to many other experimental studies, our study is solely based on human samples. It is important to describe events under baseline conditions and not only under extreme conditions using various challenges and triggers in preclinical models of asthma to demonstrate that differences between sexes may contribute to the disease. One limitation of our study is that we are looking at associations and therefore some of our conclusions are still hypothetical as no mechanistic evidence can confirm causal relationships. Additional limitations are that we only have samples from a single timepoint and that the study population is quite small. Hence, our study may not necessarily represent the general population and therefore could be considered preliminary. A higher number of participants with airway samples could have increased the statistical power and validation in an additional cohort would have strengthened the result. However, given that the let‐7 miRNAs in bronchial biopsies are strongly associated with asthma control and asthma exacerbations, they may be potential targets for modulation of treatment at the site of inflammation.

### 4.2 Perspectives and significance

While previous studies have reported decreased expression of let‐7 family members in asthma, this is the first study to evaluate sex differences in airway samples. Here we demonstrate the potential involvement of let‐7 miRNAs in asthma, and as a possible driver of the differences between male and female patients. The tendency for increased expression of let‐7 miRNA in males compared with females may contribute to the lower prevalence of asthma in males compared with females after adolescence. However, the mechanism(s) behind the regulation of let‐7 miRNAs, particularly in asthma, remains to be elucidated. Due to the nature of miRNAs, several miRNAs can target a unique mRNA whereas a single miRNA can target several mRNAs. It is also likely that let‐7 miRNAs are not the only miRNAs involved as has been demonstrated in other studies.^30^ Future studies are needed to elucidate the role of let‐7 miRNAs in cohorts of patients with severe asthma and to longitudinally follow patients through therapy over time.

## 5 CONCLUSION

Let‐7 miRNAs may act as immune modulators in asthma by inhibiting the production of IL‐13, resulting in lower levels of IL‐13 in males compared to females. These lower IL‐13 levels may also be associated with lower TSLP levels in males with asthma compared with females. Increased ST2L expression may promote the increased airway ILC2s observed in males with asthma. In contrast, increased sST2 in females may interfere with the chemoattractant function of IL‐33, resulting in fewer airway ILC2s in females with asthma compared with males. In conclusion, the let‐7 family miRNAs may exert sex disparity‐dependent immunomodulatory functions in human asthma which need further investigations.

## AUTHOR CONTRIBUTIONS

Carina Malmhäll, Kristina Johansson, Linda Ekerljung, Roxana Mincheva, Christopher McCrae, Henric Olsson and Madeleine Rådinger contributed to the study design. Carina Malmhäll, Jenny Calvén, Julie Weidner, Kristina Johansson, Patricia Ramos‐Ramírez, Emma Boberg and Madeleine Rådinger performed experiments and data analysis. Carina Malmhäll, Jenny Calvén and Madeleine Rådinger wrote the manuscript. All authors contributed to revision of the manuscript and provided intellectual input.

## CONFLICT OF INTEREST STATEMENT

Julie Weidner, Emma Boberg, Christopher McCrae and Henric Olsson are current employees and/or stockholders of AstraZeneca. Hannu Kankaanranta reports fees for lectures and consultancies from AstraZeneca, Boehringer‐Ingelheim, Chiesi Pharma, COVIS Pharma, G.S.K., M.S.D., Orion Pharma and Sanofi Genzyme outside the current study. Madeleine Rådinger reports partial funding for the current study from Astra Zeneca. All other authors declare no conflicts of interest.

## Supporting information

Supporting Information S1

Table S4

Figure S1

Figure S2

## Data Availability

The data that support the findings of this study are available on request from the corresponding author. The data are not publicly available due to privacy or ethical restrictions.
