# Supplementary material for: Sex disparity in adult asthma—A potential immunomodulatory role of let‐7 family microRNAs
Source: Clin Transl Allergy. 2025 Feb 28;15(3):e70042. doi: 10.1002/clt2.70042 (PMC11871111; doi:10.1002/clt2.70042)
Supplement: Supplementary file 1 — Supporting Information S1 [file CLT2-15-e70042-s003.docx]

**Supporting information**

**Sex disparity in adult asthma – a potential immunomodulatory role of let-7 family microRNAs**

Carina Malmhäll, Jenny Calvén, Julie Weidner, Kristina Johansson, Patricia Ramos-Ramirez, Emma Boberg, Linda Ekerljung, Roxana Mincheva, Bright Nwaru, Hannu Kankaanranta, Henric Olsson, Christopher McCrae, Madeleine Rådinger*.

*Corresponding author address

Prof. Madeleine Rådinger

University of Gothenburg

Sahlgrenska Academy

Inst of Medicine

Department of Internal Medicine and Clinical Nutrition

Krefting Research Centre

Box 424

SE-405 30 Gothenburg

madeleine.radinger@gu.se

**Methods**

*Study participants*

Study participants were either invited from the West Sweden Asthma Study (n=60; 70.6%), a population-representative longitudinal study on adult asthma and respiratory health or recruited via advertisement (n=25; 29.4%). All participants completed a self-administered questionnaire, a structured clinical interview, and a clinical examination^1^. The asthma control as determined by GINA 2006 ^2^ was used to evaluate asthma symptoms and control at the clinical interview. Level of asthma control were determined as controlled, partly controlled or uncontrolled. An asthma exacerbation was defined as a change in the treatment for a period due to illness or severity of disease. Asthma subjects had physician-diagnosed asthma and were currently on treatment with inhaled corticosteroids (ICS). Control subjects did not report asthma symptoms. Exclusion criteria were current smoking, other respiratory disease, autoimmune disease, cancer, common cold or a C-reactive protein (CRP) value >5 mg/ml. Atopy was defined as a positive skin prick test (SPT) and/or a positive Phadiatop (>0.35 kU/L). Both the SPT and Phadiatop panels consisted of allergens composed of birch, timothy, mugwort, cat, dog, horse, *Claudisporium herbarum, Dermatophagoides farina and D. pteronyssius* (ALK, Horshol, Denmark and Thermo Scientific respectively). The SPT panel also included *Alternaria (ALK)*. The SPT was considered positive with a wheal and flare reaction >3 mm for at least one allergen. Within two weeks after the clinical examination, all participants volunteered to donate blood samples (n=85), and some participants additionally volunteered to undergo bronchoscopy for sampling (n=55). All subjects gave written informed consent to a study protocol approved by the Regional Ethical Committee, Gothenburg, Sweden (no.228-14)

*Bronchoscopy sampling*

Bronchoscopy was performed at the Lung Diagnostic facility at Sahlgrenska University Hospital, Gothenburg, Sweden. Bronchial lavage was performed by instilling and retrieving 20 ml sterile pyrogene-free PBS three times. The first flush was handled as a separate sample (bronchial wash); the second and third flushes were pooled. The samples were filtered through a 70 µM cell strainer prior to centrifugation to separate cells from fluid. One tablet of proteinase inhibitor cocktail (cOmplete mini EDTA-free; Roche Diagnostics GmbH, Mannheim, Germany) was added to 10 ml aliquots of cell-free BL fluid, which were then immediately frozen at -80ᵒC until further analysis. Cells from the first wash were combined with the pooled cells from the second and third flushes and manually counted using Türk´s solution; cell viability was estimated through trypan blue exclusion. Cytospin slides were prepared and stained for differential cell count. Cells from the combined washes were seeded in 24-well plates for 90 min to remove adherent cells. Non-adherent cells were recovered and stained for flow cytometry analysis. Demographic and clinical characteristics of study participants included in bronchial lavage sampling are found in Supplementary Table 3.

Bronchial biopsies were obtained using mechanical biopsy forceps (Olympus Co. Tokyo, Japan) and cryo-equipment (Erbokryo CA, ERBE Elektromedizin GmbH, Germany) from the lateral segment of the right middle lobe bronchus. Biopsies were transported in pyrogen-free PBS on ice and snap-frozen dry within 30 min. Biopsies were kept at -80ᵒC until analysis.

*Blood sampling and peripheral blood mononuclear cells (PBMC) isolation*

Whole blood was collected for differential cell count, CRP measurements (Clinical Chemistry Laboratory, Sahlgrenska University Hospital) and serum samples. PBMCs isolated by density centrifugation using Ficoll-Paque™ PLUS (Cytiva Sweden AB, Uppsala, Sweden) were used in flow cytometry analyses.

*Flow cytometry*

Bronchial lavage cells and PBMCs were incubated with 1 mg/ml of Human IgG (Sigma, Saint Louis, Missouri, USA), followed by staining with a viability dye (Live/Dead®Fixable Aqua Dead Cell Stain, Invitrogen, Life Technologies corp, Eugene, Oregon) and antibodies to detect surface antigens: Lin cocktail (CD3, CD14, CD16, CD19, CD20, CD56, CD123, CD11c, FcεRIα), ST2/IL-33R, CD127, CD4, CRTH2/CD294.

Samples were processed on a BD FACSVerse running BD FACSuite software (BD Biosciences, San José, CA, USA). Data were analyzed with FlowJo software (Tree Star, Ashland, Oregon). Only live singlet CD45+ lymphocytes were analyzed. Lineage negative cells were defined as CD3-, CD14-, CD16-, CD19-, CD20-, CD56-, CD123-, CD11c- and FcεRI-. ILCs were defined as Lin- and CD127+. ILCs positive for either CRTH2, ST2 or the combination were considered ILC2s. Lin+ CD4+ cells were considered Th cells. CRTH2+ST2+ or CRTH2+ST2- Th cells were considered Th2 cells. Gating was determined using control samples by the fluorescence minus one (FMO) approach, i.e., controls containing all markers except the one of interest were used to set gates. Representative gating strategy is shown in Figure 1A. A table of antibodies used for flow cytometry is found in the Supplementary Table 1.

*Mediator analysis*

Human IL-33, IL-13, sST2/IL-33R and TSLP were analyzed in cell-free bronchial lavage and serum using DuoSet ELISA kits (R&D Systems, Minneapolis, MN, USA). The BD OptEIA™TMB Substrate Reagent Set (BD Bioscience, San Diego, CA, USA) or the BM Chemiluminescence ELISA Substrate (POD) (Roche Diagnostics GmbH, Mannheim, Germany) were used for signal detection. Testosterone and estradiol levels in serum samples were determined using a testosterone assay and an estradiol assay (Parameter™, R&D Systems, Minneapolis, MN, USA). Absorbance or luminescence values were determined using a Varioskan™ LUX multimode microplate reader running SkanIt™ Software (Thermo Fisher Scientific Oy, Vantaa, Finland). Values below the detection limit were set to a mean value between zero and the detection limit for each mediator analyzed.

*RNA isolation and microRNA expression analysis using NanoString and RT-qPCR*

Bronchial biopsies were homogenized and lysed in QIAzol® Lysis Reagent in GentleMACS M Tubes using a GentleMACS Dissociator (Miltenyi Biotec GmbH). Total RNA was isolated using miRNeasy microKit (Qiagen) according to the manufacturer’s protocol with the exception that Phasemaker™ tubes (Invitrogen by Thermo Fisher Scientific, Life Technologies Corp. Carlsbad, CA, USA) were utilized in the phase separation stage as described in the manufacturer’s user guide. Eluted RNA was measured using the Agilent RNA 6000 Nano kit on an Agilent 2100 Bioanalyzer running 2100 Expert Software (Agilent Technologies Inc., California, USA) or a DeNovix Microvolume Spectrophotometer DS-11FX+ (DeNovix, Inc, DE, USA).

RNA from 12 bronchial biopsies, three donors from each group, were analyzed using a targeted multiplex miRNA expression assay, nCounter® Human v3 miRNA Assay and nCounter® miRNA Sample prep kit (NanoString technologies, Seattle, Washington, USA). The panel consisted of 827 miRNAs, 5 mRNAs, and 25 internal reference controls. Hybridization and analysis were performed using the nCounter® system (NanoString technologies). Data were analyzed using the nSolver analysis software (version 4.0). Raw miRNA counts were normalized to total counts of the top 100 most highly expressed miRNAs across all samples. Negative controls were used to determine the background threshold based on calculating the average count of 7 negative controls +2 standard deviations. miRNAs with counts above the threshold were considered detected miRNAs.

RNA from 27 bronchial biopsies, including the 12 biopsies mentioned above, was reverse transcribed using 20 ng of total RNA per cDNA reaction with the miRCURY LNA RT Kit (Qiagen) according to the manufacturer’s protocol. Quantitative real-time PCR (qPCR) was performed on a CFX96 Touch Real-Time PCR Detection System (Bio-Rad Laboratories, Hercules, CA, USA) using cDNA diluted 1:40 and LNA primers for human let-7a-5p, let-7f-5p, let-7g-5p, miR-98-5p, miR-103-3p, control primer UniSp6 (miRCURY LNA miRNA PCR Assay, Qiagen) and miRCURY LNA SYBR Green PCR kit (Qiagen). The relative expression of each miRNA was calculated by the 2−ΔΔCq method and normalized to reference miR-103-3p and the mean value of healthy subjects. A table of primers with sequence, miRbase ID and Gene globe ID is found in the supplementary material (Supplementary Table 2). Demographic and clinical characteristics of study participants included in the NanoString assay and miRNA qPCR analysis respectively, are found in Supplementary Table 4.

**References**

1. Nwaru BI, Ekerljung L, Rådinger M, et al. Cohort profile: the West Sweden Asthma Study (WSAS): a multidisciplinary population-based longitudinal study of asthma, allergy, and respiratory conditions in adults. BMJ Open. 2019 Jun 19;9(6):e027808.
2. Koshak EA. Classification of asthma according to revised 2006 GINA: Evolution from severity to control. Ann Thorac Med. 2007 Apr;2(2):45-6.

**Supplementary Table 1. Antibodies for flow cytometric analysis**

| **Antigen (clone)** | **Format** | **Manufacturer** |
| --- | --- | --- |
| Lineage cocktail:CD3 (SK7), CD14 (MΦP9), CD16 (3G8), CD19 (SJ25C1), CD20 (L27), CD56 (NCAM16.2) | FITC | BD Biosciences, San Jose, California |
| CD123 (7G3) | FITC | BD Pharmingen™, BD Biosciences |
| CD11c (B-ly6) | FITC | BD Pharmingen™, BD Biosciences |
| FcεRI (AER-37) | FITC | BioLegend, San Diego, California |
| CD127 (HIL-7R-M21) | PE | BD Pharmingen™, BD Biosciences |
| CD45 (2D1) | PerCP | BD Biosciences |
| ST2/IL-33R (polyclonal Goat IgG) | APC | R&D Systems®Minneapolis, Minnesota |
| CD4 (RPA-T4) | APC-H7 | BD Pharmingen™, BD Biosciences |
| CRTH2/CD294 (BM16) | BV421 | BD Horizon™, BD Biosciences |

APC, Allophycocyanin; APC-H7, H7 conjugate of Allophycocyanin; BV421, Brilliant Violet 421; FITC, Fluorescein isothiocyanate; PE, phycoerythrin; PerCP, Peridinin chlorophyll protein.

**Supplementary Table 2. LNA primers for miRNA RT-qPCR**

| **miRNA** | **GeneGlobe ID** | **miRBase ID** | **miRNA Sequence 5’ -3’** | **Precursors genomic location** | **In silico target prediction relevant to this study (miRDB target score)** |
| --- | --- | --- | --- | --- | --- |
| hsa-Let-7a-5p | YP00205727 | MIMAT0000062 | UGAGGUAGUAGGUUGUAUAGUU | chr9, chr11, chr22 | IL13 (90) |
| hsa-Let-7f-5p | YP00204359 | MIMAT0000067 | UGAGGUAGUAGAUUGUAUAGUU | Chr9, chrX | IL13 (90) |
| hsa-Let-7g-5p | YP00204565 | MIMAT0000414 | UGAGGUAGUAGUUUGUACAGUU | Chr3 | IL13 (90) |
| hsa-miR-98-5p | YP00204640 | MIMAT0000096 | UGUGAGGUAGUAAGUUGUAUUGUU | ChrX | IL13 (90) |
| hsa-miR-103-3p | YP00204063 | MIMAT0000101 | AGCAGCAUUGUACAGGGCUAUGA | Chr5, chr20 |  |
| UniSp6 | YP00203954 | UniSp6 | CUAGUCCGAUCUAAGUCUUCGA |  |  |

All LNA primers from Qiagen

**Supplementary Table 3. Demographic and clinical characteristics of study participants included in bronchial lavage sampling**
