# Supplementary material for: Sex disparity in adult asthma—A potential immunomodulatory role of let‐7 family microRNAs
Source: Clin Transl Allergy. 2025 Feb 28;15(3):e70042. doi: 10.1002/clt2.70042 (PMC11871111; doi:10.1002/clt2.70042)
Supplement: Supplementary file 2 — Table S4 [file CLT2-15-e70042-s001.docx]

**Supplementary Table 4.** **Demographic and clinical characteristics of study participants included in NanoString assay and miRNA qPCR analysis respectively**
