# Supplementary figures and images for: Sex disparity in adult asthma—A potential immunomodulatory role of let‐7 family microRNAs
Source: Clin Transl Allergy. 2025 Feb 28;15(3):e70042. doi: 10.1002/clt2.70042 (PMC11871111; doi:10.1002/clt2.70042)

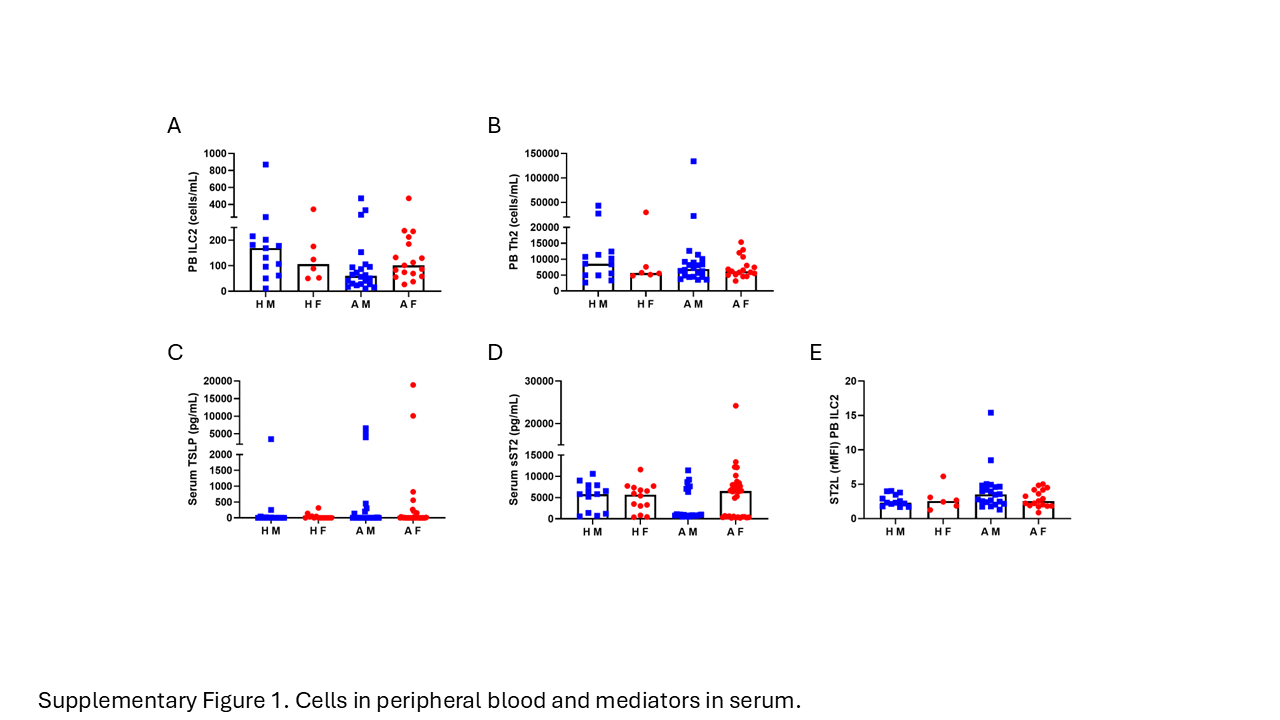

Supplement: Supplementary file 3 — Figure S1 [file CLT2-15-e70042-s004.TIF]

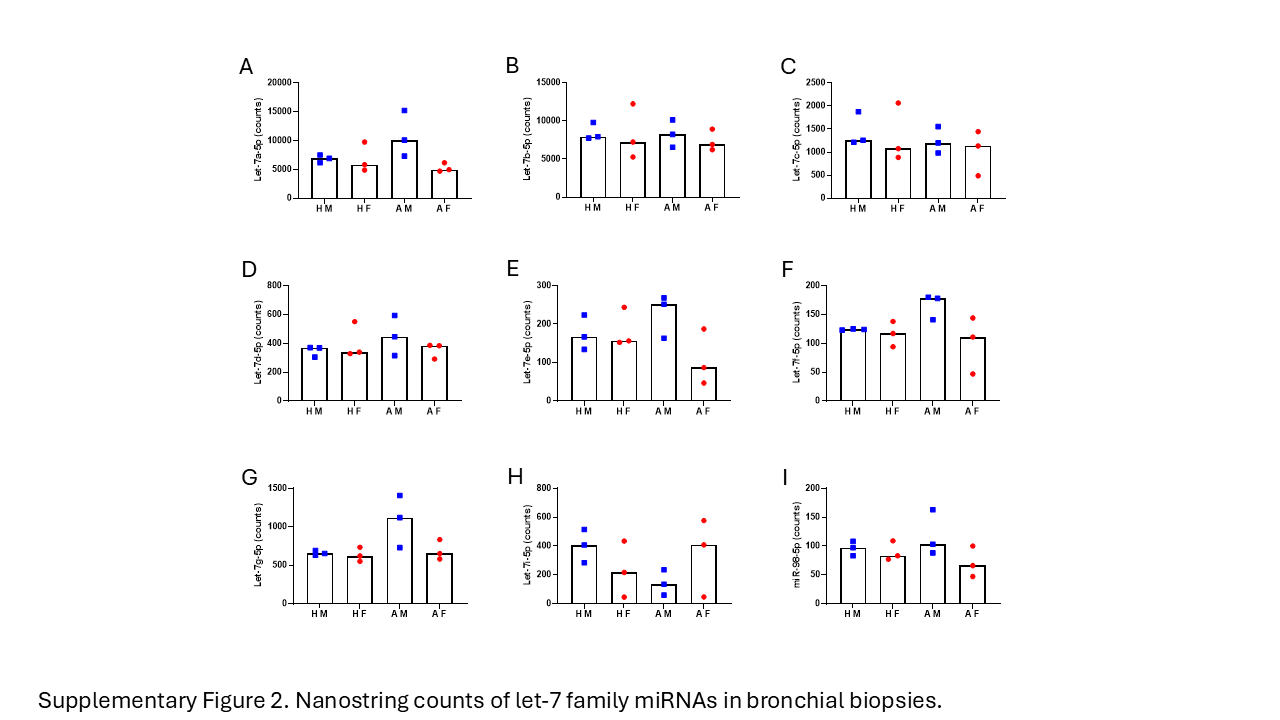

Supplement: Supplementary file 4 — Figure S2 [file CLT2-15-e70042-s002.TIF]
